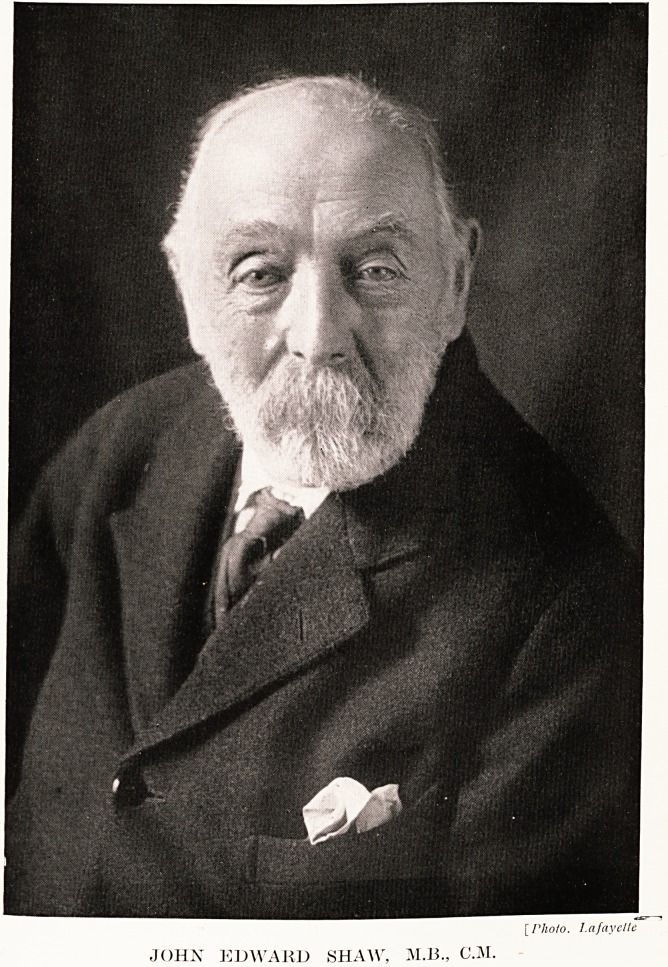# J. E. Shaw

**Published:** 1939

**Authors:** 


### Obituary

### JOHN EDWARD SHAW, M.B., C.M.

THE death of Dr. Shaw on 9th March has taken from us not only the doyen of the Royal Infirmary Staff and a man of the widest culture and interests, but a picturesque personality, long well-known and well-loved. He was born in Bath in 1848 and went to school there: although as a boy he was considered delicate, yet when he entered the Bristol Medical School in 1866 he used to get up every morning at six in order to get from Bath to his classes in Bristol punctually. In 1869 he went to Edinburgh to complete his course, and qualified there in 1872. He was House Physician at the Edinburgh Royal Infirmary, a Fellow of the Edinburgh Botanical Society and President of the Royal Medical Society of Edinburgh. In 1875 he returned to Clifton and two years later was elected Physician to the Bristol Royal Infirmary, a post that he held for thirty years: in 1907 he was appointed Consultant Physician. He was adviser in Lunacy to the Bristol Magistrates from 1879 to 1911, and again from 1917 to 1921: and Professor of Medicine and Lecturer in Practical Medicine and Materia Medica at the University College from 1895 to 1905. He was also Physician to the Bristol Blind Asylum. He was President of our Society in 1897; and in 1894 Vice-President of the Section for Diseases of Children at the Annual Meeting of the British Medical Association.

Dr. Shaw always took an intense personal interest in his patients, and so loved his work that he could not resolve to retire from practice: he was in fact seeing patients right up to the very end of his long life. Reference to our index will show how many and varied were his contributions to our pages. As a teacher he was greatly valued. A former student of his writes: "I think the outstanding characteristics which I recall were his courtesy and politeness. There was a definite drawback in clerking for Shaw, in that he always treated you as if you knew as much about a case or subject as he did and therefore he would not insult your intelligence by

[Photo. Lafayette

JOHN EDWARD SHAW, M.B., C.M.

instructing you. He was an extraordinarily good mimic, especially of various functional nervous disorders, and his lectures on this topic were the most interesting and impressive that I recall during my time as a student; because Shaw's antics, as he demonstrated the various spasms, gaits, fits, etc., brought the subject home to his audience in a very practical way."

Apart from his work, Shaw's interests were of the widest. He was one of the original members of the Bristol and Gloucestershire Archæological Society. He took a keen interest in the theatre, and was President of the Medical Dramatic Club in 1901–02. All through his life he was an enthusiastic gardener, and for many years habitually wore an orchid of his own growing. For years he was a breeder of dachshunds, a hobby that earned him his nickname of "Doggy" Shaw. He also bred and showed fancy poultry. He was a great amateur of horseflesh, and regularly drove a carriage with a striking pair of black horses; he bred polo ponies, until the restrictions imposed by the Great War terminated that activity. When to this catholicity of interests we add a profound knowledge of his professional work, and a kindness of heart universally acknowledged, it is easy to understand how great was the influence Shaw exercised for so many years in the life of our school and city.

## Figures and Tables

**Figure f1:**